# Changes in fear-associated learning task brain activation over the COVID-19 pandemic period: a preliminary longitudinal analysis

**DOI:** 10.3389/fpsyt.2023.1239697

**Published:** 2023-11-22

**Authors:** Claire Popovich, Aaron S. Grau, Chia-Hao Shih, Neejad T. Chidiac, Adrian Zhou, Xin Wang, Hong Xie

**Affiliations:** ^1^Department of Psychiatry, The University of Toledo, Toledo, OH, United States; ^2^Department of Emergency Medicine, The University of Toledo, Toledo, OH, United States; ^3^Department of Neurosciences, The University of Toledo, Toledo, OH, United States

**Keywords:** COVID-19, mental health, posttraumatic stress symptom, functional magnetic resonance imaging (fMRI), fear conditioning

## Abstract

**Background:**

The COVID-19 pandemic has had profound impacts on people worldwide. Previous studies have shown that fear learning, extinction, recall, and contextual information processing involve the activation of emotion and sensory brain systems, which can be modified. However, it remains unclear whether brain functions associated with these processes have been altered over the pandemic period.

**Methods:**

We compared pre- and peri-pandemic brain activation during a fear-associated learning task (FALT) using previously collected data. The participants were divided into two groups: the pandemic group (*n* = 16), who completed a baseline FALT before the pandemic and repeated the task during the pandemic, and the non-pandemic group (*n* = 77), who completed both sessions before the pandemic began.

**Results:**

Compared with the non-pandemic group, the pandemic group exhibited significant decreases in brain activation from baseline to follow-up assessments, including activation in the brainstem during early fear learning, the posterior thalamus/hippocampus during late extinction, and the occipital pole during late recall phases for contextual processing. Furthermore, activations associated with retrieving safety cues were reduced in the posterior cingulate, premotor, and calcarine cortices during the early recall phase, and activations associated with retrieving dangerous cues decreased in the occipital pole during the late recall phase. Additionally, correlations between decreased activation and elevated posttraumatic stress symptoms were observed.

**Conclusion:**

These findings suggest that activations associated with processing low arousal contextual information, safety cues, and extinguished fear cues decreased during the pandemic. These changes in brain activation may have contributed to the increase in mental health disturbances observed during this time.

## 1 Introduction

COVID-19 introduced the worst global pandemic in a century, with more than 622 million cases and 6.55 million confirmed deaths (1). Policies such as lockdowns, social distancing, and quarantining reduced transmission but caused severe social and economic disruptions, such as social isolation, physical confinement, disruption of education, widespread supply shortages, unemployment, and global recession. Additionally, dramatic increases in mental health conditions, such as anxiety, depression, and posttraumatic stress disorder (PTSD), have been reported worldwide (2–6). Considering that a recent study showed pandemic-related anxiety is associated with an impairment of fear learning and with the generalization of fear to ubiquitous stimuli (7) and that previous research showed that disruptions in the learning and processing of fear can underlie psychiatric symptoms (6, 8, 9), it is likely that the abnormal processing of fear during the pandemic may have contributed to increased mental health disorders.

In addition to these initial behavioral findings that link fear learning functions with mental health symptoms caused by the pandemic, the study of brain activation to fear learning and memory during the pandemic may provide a greater understanding of the neurobiological mechanisms that underlie pandemic-associated increases in psychiatric disturbances in the general public. Previous fMRI studies that used the fear-associated learning task (FALT) have successfully examined brain activation connected to the learning, extinction, and retention of fear-evoking and safety cues, as well as the contextual information associated with these cues (9). Using FALT, alterations in brain activation have been reported in patients with multiple psychiatric disorders, including PTSD, general anxiety disorder (GAD), specific phobia, and schizophrenia (9). Alterations in activations associated with fear acquisition, extinction learning, extinction recall, and context processing have been found in the insula, anterior cingulate cortex (ACC), amygdala, thalamus, midbrain and hippocampus, and medial prefrontal cortex (mPFC) across multiple disorders (10–12). Furthermore, associations between brain function alterations and disorder-specific symptom severity have been reported (10, 13, 14). These findings suggest links between impairments in fear-associated learning brain functions and psychiatric disturbances. Yet, whether pandemic experiences could have altered fear-associated learning functions and the underlying brain processing remains unanswered.

Given the unexpected onset of the COVID-19 pandemic, it was not feasible to plan well-designed studies comparing pre- and peri-pandemic mental health symptoms and FALT-related brain function in the same individuals. Therefore, we leveraged data collected from a longitudinal study, which included participants who completed 1-year follow-up assessments after experiencing life-threatening traumatic events before the pandemic, as well as another group of participants who experienced the pandemic during the 1-year follow-up period. In this case–control longitudinal analysis, we aimed to investigate changes in brain activation related to fear-associated learning functions between the two groups and their associations with posttraumatic stress symptoms. It should be noted that our focus is not on the impacts of COVID-19 infection on brain function, but rather on considering COVID-19 as a unique, stress-inducing event for the participants in this investigation.

## 2 Materials and methods

### 2.1 Participants

Data from 93 participants (34.0 ± 10.9 years; 64.5% female) who completed both initial and follow-up assessments from 2017 to 2021 were included in the present study. The original longitudinal study aimed to investigate early and longitudinal brain changes associated with PTSD development after trauma exposure (15). In brief, adult trauma survivors were recruited from local hospital emergency departments (EDs) within 48 h after experiencing a traumatic event and followed up for 1 year. Participants were excluded if they: (a) were under the influence of alcohol or drugs at the time of the traumatic event, (b) had major body injuries, (c) had moderate-to-severe traumatic brain injury, (d) had major medical illness affecting general health, (e) were diagnosed with major psychiatric disorders including PTSD, or (f) had conditions that precluded MRI or assessment procedures (e.g., metallic objects in the body). All participants completed initial assessments including MRI scans within 2 weeks following their traumatic experience, as well as follow-up assessments including MRI scans ~1 year after the trauma. Among them, 16 participants completed follow-up assessments after March 2020 and were considered as the pandemic group because they completed the follow-up assessments over the pandemic period while the social distancing policy was in place; three of the 16 participants reported being infected by COVID-19, but all of them were symptom-free during the follow-up assessments. The remaining 77 participants completed follow-up assessments before March 2020 and were considered as the non-pandemic group because they did not experience the pandemic during the follow-up period. The study was approved by The University of Toledo's Institutional Review Board, and all participants provided written informed consent.

### 2.2 Mental health condition

Participants' mental health conditions were evaluated with surveys and interviews during the study period. The Mini-International Neuropsychiatric Interview (MINI) (16) and the Clinician-Administered PTSD Scale (CAPS) for DSM-V (17) were administered by trained clinical psychologists at a 1-year follow-up assessment. The MINI is designed as a brief structured diagnostic interview for the major psychiatric disorders, whereas the CAPS is a 30-item, structured interview that assesses the intensity and frequency of PTSD symptoms. Both of the instruments have shown adequate reliability and validity (16–18). Additionally, participants' posttraumatic stress symptoms were assessed with the PTSD Checklist (PCL) for DSM-V (19) at both initial and follow-up assessments. The PCL consists of 20 items that match the diagnostic symptom criteria for PTSD. Participants were instructed to rate symptoms on a 5-point Likert-type scale (i.e., 0 = “Not at all” to 4 = “Extremely”). The PCL-5 has shown adequate psychometric properties (20, 21). It should be noted that both CAPS and PCL-5 assessments were anchored to the index trauma that qualified participants for the study and that none of the participants reported additional trauma, including the COVID-19 pandemic, as a severe trauma that met criterion A for PTSD diagnosis.

### 2.3 Brain imaging

Imaging acquisition and processing. Participants were scanned on a 3T GE Signa HDxt MRI scanner with an eight-channel head coil. Functional MRI scans were collected with a T2^*^-weighted, echo-planar/gradient echo pulse sequence (TR = 2000 ms, TE = 30 ms, FA = 90°, phases = 139, FOV = 240 × 240 mm, matrix = 64 × 64, slice thickness = 3.5 mm). A T1-weighted gradient echo overlay (TR = 7.864 ms, TE = 2.98 ms, TI = 650 ms, FA = 9°, FOV = 240 × 240 mm, matrix = 256 × 256, number of slices=36, slice thickness = 3.5 mm) and a high-resolution 3D FSPGR structural MRI image (TR = 7.836, TE=2.976, FA = 9°, FOV = 256 × 256 mm, matrix = 256 × 256, number of slices = 164, slice thickness = 1 mm) were collected for registration (22).

All fMRI images were processed with FSL version 5.0.10 using standard procedures (23). All scans were visually inspected for imaging quality, and problematic images were excluded. Activations associated with conditioned stimulus plus (CS+), conditioned stimulus plus extinguished (CS+E), conditioned stimulus plus unextinguished (CS+U), conditioned stimulus minus (CS-), and context were defined using fixation as a baseline using a general linear model (GLM) according to time-series data acquired from E-Prime. The activation maps of individual participants were averaged and registered to the standard 2-mm Montreal Neurological Index (MNI) template, and the initial contrast image was subtracted from the corresponding follow-up contrast image to define changes over time. Finally, group comparisons of changes over time between the pandemic and non-pandemic groups were performed while controlling for age, sex, and interval between MRI sessions in FEAT's mixed-effect model. Multiple comparison correction was applied using FSL GRF-theory-based cluster thresholding at voxel-wise z threshold > 3.1 (i.e., *p* < 0.001, one-tailed) and a cluster-wise p threshold of 0.05, for both the positive and negative contrast directions. The FEAT-related activations were then extracted from the contrast of parameter estimate (COPE) images of tasks. FEAT query was used to extract mean percentage COPE values.

Fear-associated learning task (FALT). During MRI scans, participants performed FALT modified from Garfinkel et al. (24) over 2 days (Supplementary Figure 1). FALT involved four phases: Habituation, Fear Acquisition, Fear Extinction (day 1), and Extinction Recall (day 2). Among these phases, Acquisition, Extinction, and Recall phases were split into two equal runs to study early and late activation in each phase based on previous research (24). During the Habituation, 12 combinations of context (i.e., rooms) and CS (i.e., color lights) were presented without an aversive unconditioned stimulus (US) to ensure that participants were familiar with stimuli and contexts and to demonstrate that there were no pre-learning differences between responses to the cures. In the Acquisition, one dangerous context (e.g., office) was presented for 2–7 s, followed by a CS [e.g., pink, yellow, or blue light) for an additional 3 s. An aversive US (burst of loud white noise (100 dB, 500 ms)] was delivered at 60% contingency (12 of 20 trials of each color light) after both yellow and blue lights to establish CS+ stimuli. The remaining CS (i.e., pink light) was presented without an aversive US in 20 trials to establish the CS- stimulus. The 20 CS- trials were interleaved with 40 CS+ trials. In the Extinction with a safety context (e.g., library) that followed immediately after the Acquisition, one kind of CS+ (e.g., yellow light) was presented in the absence of the US in 20 trials to form CS+E and interleaved with 20 CS- trials. For the Extinction Recall on day 2, testing was done in the safety context (e.g., library). Participants were again shown the CS- (pink light, 20 trials) interleaved with CS+ that was extinguished (CS+E, yellow light, 20 trials) and unextinguished (CS+U, blue light, 20 trials) on day 1. The intertrial interval (ITI) in all phases was a white fixation cross on a black background, which jittered for 12–18 s. In the follow-up MRI session, stimuli and ITI timings were identical to those used in the initial MRI session, but the contexts and CS were changed to preserve task novelty. Stimuli were presented in the MRI scanner with a goggle system (NordicNeuroLab, Bergen, Norway). E-Prime was used for stimulus presentation and response collection, with an MR-compatible response device (Psychology Software Tools, Inc., Sharpsburg, PA). Participants were asked to rate the likelihood of US during the CS presentation on a 5-point Likert-type scale.

Fear acquisition was evaluated with psychophysiological reactions using skin conductance reactions (SCRs) derived from event-related electrodermal responses (EDRs). EDR was recorded and processed using BIOPAC AcqKnowledge software and established procedures (24). An SCR was calculated by subtracting the baseline EDR level during the context presentation immediately prior to CS onset from the maximum EDR level during the CS presentation. An SCR < 0.01 μS was scored as zero. The data were square root transformed.

### 2.4 Statistical analysis

The demographic characteristics and mental health conditions of participants were analyzed across the pandemic and non-pandemic groups using independent *t*-tests or chi-square tests as appropriate. A factorial ANOVA was used to assess the impact of time (i.e., baseline vs. follow-up), phase (i.e., early vs. late), stimulus type, and group affiliation on both SCR and US expectancy. Partial correlation analyses were conducted to investigate the relationships between changes in FALT-related brain activations and in PCL-5 total scores over time, while controlling for variables such as age, sex, and scan interval. Moderation analysis further explored whether group status influenced the relationship between FALT-related brain activation and PCL-5 total scores. In our dataset, all variables adhered to the assumption of normal distribution, with the exceptions of SCR and US expectancy. Preliminary analyses, including the Shapiro–Wilk test and visual inspections of Q-Q plots, confirmed these deviations from normality. Despite these violations, the statistical methods employed in this study pertinent to SCR and US expectancy data were robust to such deviations. Corrections for multiple comparisons were made using the false discovery rate (FDR) method. All statistical analyses were performed using SPSS version 27.0 (IBM Corp, Armonk, New York, USA). The level of statistical significance was set at α = 0.05, a two-tailed test.

## 3 Results

### 3.1 Participant characteristics

Participants' demographic, mental health, and other information are summarized in Table 1. No differences in age, sex, incidences of mental health conditions, and PCL-5 scores changes were found between the pandemic and non-pandemic groups (all *p* > 0.05). However, the interval between the two MRI sessions was significantly longer in the pandemic group (549.63 ± 128.48) than in the non-pandemic group (372.92 ± 10.17). Based on this finding and previous research (25), age, sex, and scan interval were included as covariates in subsequent analyses to mitigate potential confounding effects.

**Table 1:** Demographics and mental health conditions in the pandemic and non-pandemic groups.

	**Pandemic**	**Non-pandemic**	**Statistics**
*N*	16	77	
Age (years)	33.19 ± 10.48	34.17 ± 11.11	*t*(91) = 0.32, *p* = 0.75
Sex (Female %)	75.0 %	62.3 %	χ^2^(1) = 0.93, *p* = 0.34
Scan interval (days)	549.63 ± 128.48	372.92 ± 40.17	*t*(91) = 10.83, *p < * 0.05
**Race**			
Black/African American	7	42	χ^2^(2) = 0.84, *p* = 0.66
White	8	29	
Other	1	6	
**Mental health conditions** ^a^			
**Baseline**			
GAD	4/10	16/56	χ^2^(1) = 0.26, *p* = 0.61
MDD	4/10	12/60	χ^2^(1) = 1.10, *p* = 0.26
SUICIDAL	0/14	9/62	χ^2^(1) = 1.99, *p* = 0.16
PTSD	6/5	21/43	χ^2^(1) = 1.92, *p* = 0.17
**Follow-up**			
GAD	0/14	10/64	χ^2^(1) = 1.00, *p* =0.32
MDD	2/13	11/63	χ^2^(1) = 0.23, *p* =0.88
SUICIDAL	1/14	6/68	χ^2^(1) = 0.36, *p* =0.85
PTSD	4/11	25/51	χ^2^(1) = 0.03, *p* =0.87
PCL-5 changes	−23.56 ± 19.61	−20.92 ± 21.59	*t*(91) = 0.47, *p* = 0.64

GAD, generalized anxiety disorder; MDD, major depressive disorder; SUICIDAL, suicidal ideation/behavior; PTSD, posttraumatic stress disorder; PCL-5, PTSD Checklist for DSM-V.

a Mental health conditions were presented as positive/negative cases. Sample sizes were reduced due to missing MINI and/or CAPS interview data.

### 3.2 Skin conductance response

In the ANOVA, evidence of successful fear acquisition was observed in both the baseline and follow-up sessions. This was indicated by elevated skin conductance responses (SCRs) associated with the conditioned stimulus plus (CS+) when compared to the conditioned stimulus minus (CS–) during the Acquisition phase (all *p* < 0.05). No other statistically significant findings were detected in either the Extinction or Recall phases (all *p* > 0.05). It is important to note that a substantial portion of the SCR data was missing due to technical difficulties.

### 3.3 Behavioral US expectancy during FALT

Supplementary Figure 2 illustrates the US expectancy across group, time, and stimulus on three FALT phases separately. The main effects of the CSs were significant in all phases. *Post-hoc* analysis revealed higher US expectancy in both CS+U and/or CS+E than CS- in all phases. However, no differences between CS+U and CS+E in the Recall phase were observed. In addition, the main effect of the group also reached statistical significance in the early extinction phase, *F*_(1,86)_ = 6.17, *p* < 0.05, partial η^2^ = 0.07, where the pandemic group showed higher US expectancy than the non-pandemic group. Furthermore, in the late recall phase, a three-way interaction between time, stimulus, and group was statistically significant, *F*_(2,172)_ = 3.65, *p* < 0.05, partial η^2^ = 0.4. Follow-up analyses indicated that this three-way interaction was due to the finding that the US expectancy of CS + U decreased over time in the pandemic group but not in the non-pandemic group.

### 3.4 Brain activations associated with FALT

#### 3.4.1 Acquisition

Changes over time in activation associated with processing a dangerous contextual environment during the early acquisition phase differed significantly between the pandemic and non-pandemic groups in a cluster of the brainstem (Table 2, Figure 1A). Contextual processing activation decreased in the pandemic group over the study period, but slightly increased in the non-pandemic group (Supplementary Figure 3A). No other differences in FALT-related brain activation during early or late acquisition phases were found between the two groups.

**Table 2:** Differing changes in brain activation over time during FALT between the pandemic and non-pandemic groups.

**Brain region**	**Task condition**	**Peak Z score**	**Peak coordinate**	**Number of voxels**
***Early acquisition***				
Brainstem	Dangerous context	−4.35	10, −38, −28	533
***Late extinction***				
R Superior LOC	Safe context	−3.78	26, −58, 52	300
L Superior LOC	Safe context	−4.26	−24, −70, 54	209
L Thalamus^*^	Safe context	−4.00	−8, −30, 6	220
***Early recall***				
R PCC	CS-	−4.23	2, −20, 34	293
L Calcarine	CS-	−4.27	−12, −78, 10	180
R Premotor	CS-	−4.25	4, 28, 56	179
***Late recall***				
R Occipital pole^#^	CS+	−4.62	20, −100, 10	257
R Occipital pole^#^	Safe context	−4.95	22, −98, 8	837

* This significant cluster spanned to left posterior hippocampus (local maxima (-16, −38, 0), z score = 3.58);

# indicates this cluster survived between contrast multiple comparisons with Bonferroni correction; FALT, fear-associated learning task; L, left; R, right; CS+, conditioned stimulus with dangerous cue; CS-, conditioned stimulus with safe cue; LOC, lateral occipital cortex.

**Figure 1:**
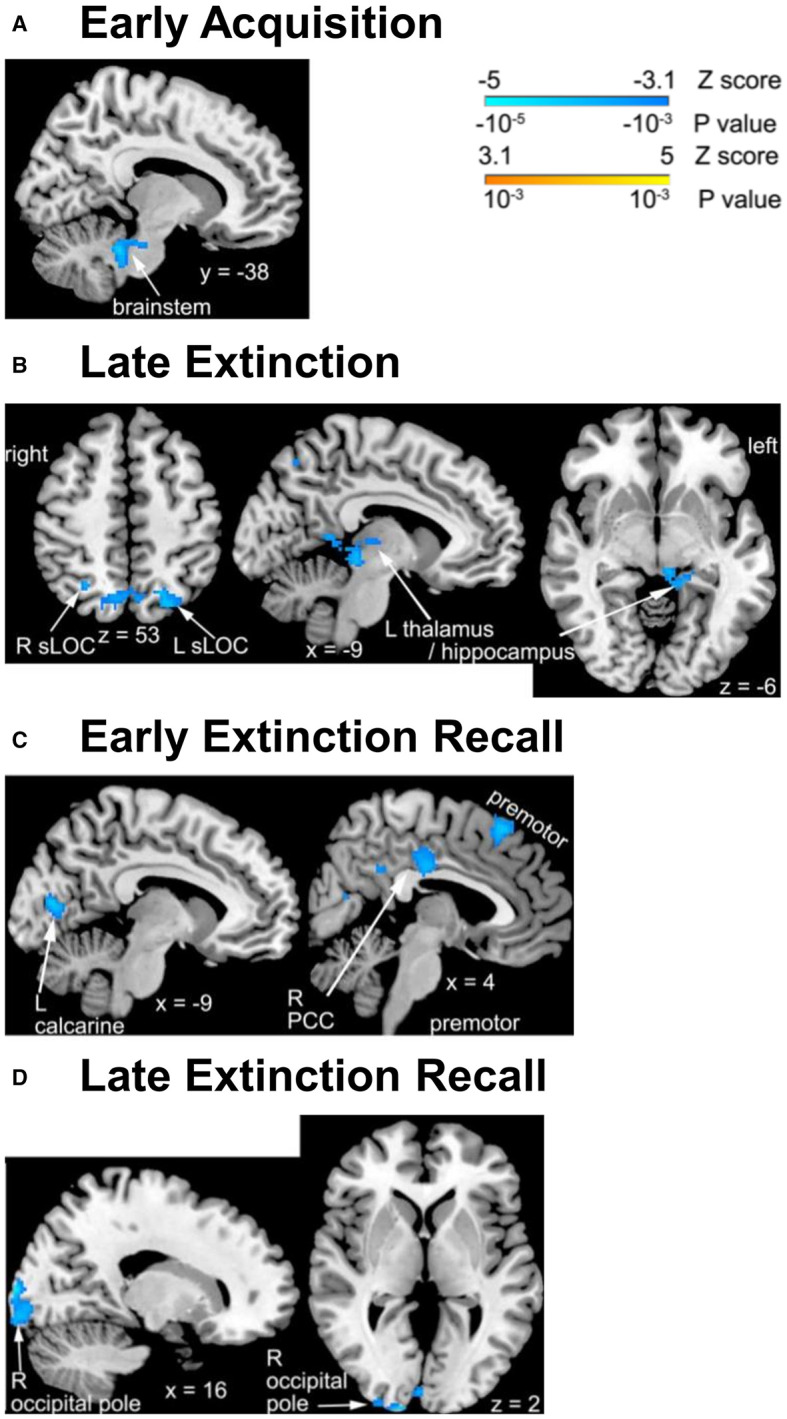
Group differences in changes in FALT activation over time across brain regions. **(A)** Significant activation difference in the brainstem for dangerous context during early fear acquisition phase; **(B)** safe context during late extinction phase; **(C)** CS− during early recall phase; **(D)** CS+ (left) during and safe context (right) during late recall phase. The clusters with significant differences between groups are illustrated on the brain images. R, right; L, left; sLOC, superior lateral occipital cortex.

#### 3.4.2 Extinction

Changes over time in activation associated with processing a safe contextual environment were significantly different between the pandemic and non-pandemic groups in the bilateral superior lateral occipital cortex (sLOC) and a cluster spanning the left posterior thalamus and posterior hippocampus during the late extinction phase (Table 2, Figure 1B). Contextual processing activation decreased in the pandemic group, but slightly increased in the non-pandemic group, over the study period in all three clusters (Supplementary Figure 3B). No other differences in FALT-related brain activation during early or late extinction phases were found between the two groups.

#### 3.4.3 Recall

Changes over time in activation associated with CS- during the early recall phase were significantly different between the pandemic and non-pandemic groups in the right posterior cingulate cortex (PCC), left calcarine cortex, and right premotor cortex (Table 2, Figure 1C). The activation associated with CS- decreased in the pandemic group but had only slight changes in the non-pandemic group over the study period in all three clusters (Supplementary Figure 3C).

Additionally, during the late recall phase, changes over time in activation associated with CS+ (combining CS+E and CS+U) were significantly different between the pandemic and non-pandemic groups in the right occipital pole (Fig 1D left). Activation associated with CS+ decreased in the pandemic group, but only had trivial changes in the non-pandemic group over the study period (Supplementary Figure 3D left). In addition, changes over time in activation associated with processing the safe contextual environment were significantly different in the right occipital pole between the pandemic and non-pandemic groups (Figure 1D right). Contextual processing activation decreased more in the pandemic group than in the non-pandemic group over the study period (Supplementary Figure 3D right). No other differences in FALT-related brain activation during early or late recall phases were found between the two groups.

### 3.5 Relationships between changes in brain activation and posttraumatic stress symptoms during pandemic

**Figure 2:**
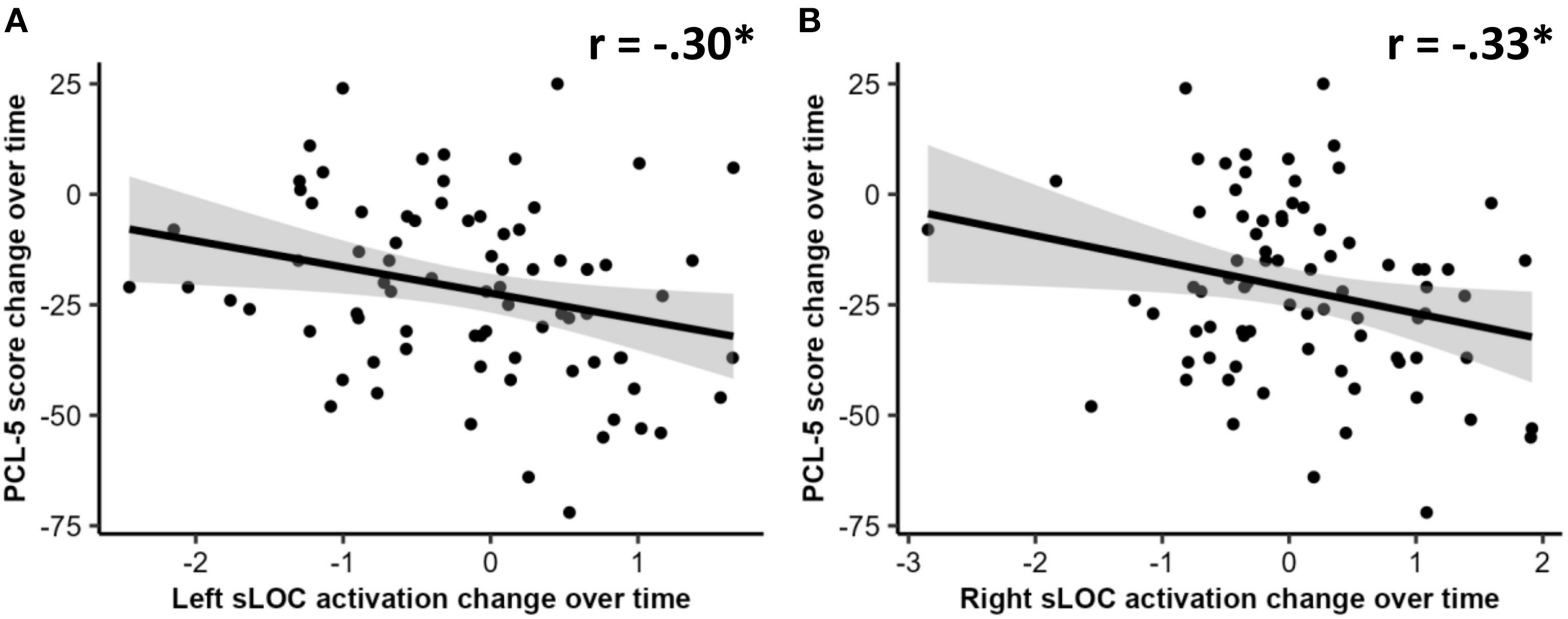
Correlations between changes over time in: **(A)** left superior lateral occipital cortex (sLOC) activation change over time and PTSD checklist for DSM-5 (PCL-5) scores; and **(B)** right sLOC activation change over time and PCL-5 scores. An asterisk (*) indicates a significant correlation at *p* < 0.05 level.

After controlling for age, sex, and scan interval, changes in activation associated with safe context during the late extinction phase in the left sLOC were negatively correlated with changes in PCL-5 scores (*r* = −0.30, *p* < 0.05, FDR-corrected). A similar negative correlation was found between changes in activation associated with safe context during the late extinction phase in the right sLOC and changes in PCL-5 scores (*r* = −0.33, *p* < 0.05, FDR-corrected). Additionally, moderation analyses indicated that the associations of these two changes of activation with changes in PCL-5 scores were not contingent upon group status (*p* < 0.05 for both interaction terms). These two correlations are visualized in Figure 2. No other significant correlation was found between FALT-associated brain activation and PCL-5.

## 4 Discussion

The present study aimed to provide initial insights into the changes in fear-associated learning brain functions over the pandemic period and their association with posttraumatic stress symptoms. Although we did not observe any differences in mental health conditions between the pandemic and non-pandemic groups, we found differences in change over time of fear-associated learning brain activation comparing the two groups. Specifically, the pandemic group showed decreased activation that (a) associated with contextual processing in the brainstem during early acquisition, in the bilateral sLOC and thalamus during late extinction, and in the occipital pole during late recall phases, (b) associated with CS- in the PCC, premotor cortex, and calcarine cortex during the early recall phase, and (c) associated with CS+ in the occipital pole during late recall phase, compared with the non-pandemic group. These reductions in fear-associated brain activation over time suggest the COVID-19 pandemic had specific influences on brain emotion-related functions. In addition, negative correlations over time of bilateral sLOC activation with PTSD symptom severity were also found. While we acknowledge that interpretations of the findings carry a degree of speculation due to the absence of direct measures of pandemic experiences, we also recognize the strength of our study design. The inclusion of a unique non-pandemic control group and the utilization of pre-pandemic baselines from the same participants lend a specific context to the observed differences between groups. This specificity pertains to the distinctive nature of the pandemic period, even considering the potential variations in individual pandemic experiences (26).

The findings of longitudinal reductions in activations in the subcortical regions and primary visual cortex suggest that pandemic experiences reduced contextual encoding and retrieval at early levels of the visual sensory processing pathway. First, the brainstem plays roles in sensorimotor and visceral integration between the cerebrum and the body (27). The reticular activating system (RAS) within the brainstem also plays a key role in facilitating and maintaining attention and conscious perception of stimuli (28). A reduction in brainstem activation mediated attention to context during fear learning, which may indicate impaired fear learning functions. Additionally, the thalamus, including dorsal medial and pulvinar nuclei, has been reported to be involved in context conditioning of dangerous vs. safe contexts (29). Furthermore, human neuroimaging studies suggest the posterior hippocampus is active during encoding contextual information (11), and impaired contextual processing on the encoding of dangerous cues in the hippocampus was reported in PTSD patients (30). On the other hand, the primary visual cortex in the occipital pole processes visual inputs and provides visual information for higher level cognitive functions. The superior LOC is involved in visuomotor function and is part of the visual attention network (31, 32). A reduction in visual attention activation to context in the LOC may impair attention to surroundings, which, in turn, may cause negative thinking and emotion numbing. Previous studies reported that contextual processing also involves the amygdala and medial prefrontal cortex (33, 34), which was not seen in the current study. It is plausible that the pandemic experiences did not impact the emotional and cognitive processing of contextual information in these brain regions. Interestingly, bilateral superior LOC activation changes were negatively associated with changes in PTSD symptom severity from pre-pandemic to peri-pandemic, suggesting that alterations in contextual processing could be related to elevated stress symptoms during the pandemic. Although these findings cannot prove causality between FALT-related brain function alterations and mental health symptoms, it is plausible that brain activation changes may mediate relationships between pandemic experiences (e.g., anxiety) and behavioral changes in fear learning.

The current study also observed decreased activation that was associated with processing CS– in the PCC, premotor area (BA 6), and calcarine cortex during early recall in the pandemic group. The PCC and calcarine cortex have been considered as parts of the safety signal processing network that is more active during learning CS– than CS+. In addition, the PCC is part of the limbic system and plays an important role in emotion processing, particularly internal feeling/sense of self and reward (35). On the other hand, the calcarine cortex is a visual region involved in visual memory, mental imagery (36), and cross-modal processing of visual and auditory information (37). Furthermore, the premotor cortex is involved in locomotion and planning voluntary movement (38) and has connections with occipital and subcortical visual processing regions (39, 40). A previous study reported that the premotor cortex is less active during conditioning of CS– vs. CS+ (41). Our findings of reductions in CS- activations in PCC, premotor cortex, and calcarine cortex suggest that pandemic experiences may reduce processing, memory, and top-down control of visual processing during retrieval of safe cues.

The pandemic group also showed decreased primary visual cortex activation associated with CS+ during the late recall phase. This finding is in line with an fMRI investigation of fear-associated learning in individuals with anxiety disorder, whereas the authors reported anxiety symptoms associated with hippocampal, frontal, visual, insular, and ACC activation, which involve contextual processing, safety cue learning and renewal, and fear cue learning and retrieval (42).

Overall, we observed that the pandemic group showed a reduction over time of brain activations associated with FALT across different phases and brain regions. While we acknowledge that the brain regions identified in our current study may not conventionally align with fear learning tasks, it is important to note that a number of studies have highlighted instances of modified context and sensory processing within fear-related fMRI tasks [see (11, 43) for reviews]. We speculated that social isolation and physical confinement secondary to quarantine measures during the pandemic may have driven these FALT-related brain activation alterations. The brainstem RAS consists of the locus coeruleus, raphe nucleus, tuberomammillary nucleus, and the lateral and dorsal pedunculopontine tegmentum (44). The RAS diffusely transmits cholinergic and adrenergic neurotransmission through the thalamus to the cerebral cortex to maintain arousal levels in these regions of the brain (27). Sensory stimulation from peripheral nerves drives brain RAS activity, and deprivation of sensory inputs by isolation and confinement during the pandemic may have lowered RAS activity and related arousal. This raises the possibility that pandemic isolation and confinement may have reduced the sensory processing of low arousal contextual, safety cues, and extinguished fear cues. Previous studies from polar exploration and spaceflights suggest that social isolation and confinement may reduce brain reactivity to sensory inputs and decrease hippocampus volume (45–47). Further studies on the mechanisms for how pandemic experiences influence fear-associated learning functions are warranted.

Regarding behavioral US expectancy, we found significant main effects of conditioned stimuli on unconditioned stimulus (US) expectancy across all phases, and notably, higher US expectancy in the pandemic group during the early extinction phase. A unique three-way interaction in the late recall phase further revealed dynamic changes in the pandemic group's US expectancy over time. In contrast, Harnett et al. (48) found no such main effects but identified a significant stimulus-by-group interaction, suggesting that trauma exposure could modulate US expectancy. The divergent findings between the two studies highlight the complex interplay of variables, including pandemic-related stress and study duration, that shape US expectancy. These discrepancies warrant further research to reconcile the observed differences.

The current study reported preliminary findings of FALT-related brain activation reductions over the pandemic period and their associations with posttraumatic stress symptoms. Yet, caution should be taken when interpreting these findings because the original longitudinal study was not specifically designed to investigate the effects of pandemic experiences. For example, although we did not observe differences between the pandemic and non-pandemic groups on their mental health conditions, it should be noted that all study participants were recruited due to trauma exposure and some subjects had developed PTSD prior to the pandemic. In addition, the difference that occurred in scan interval comparing the two groups was not desired and can only be adjusted statistically. Furthermore, we did not collect information regarding pandemic experiences directly but only administered instruments that assess psychological symptoms in general or in relationship to trauma exposure. This had the effect of limiting our understanding of the influence of pandemic experiences on changes in FALT-related brain function, behavioral symptoms, and their associations. However, it should be noted that the observed activation changes most likely did not directly result from virus infection because no participants reported active or residual COVID symptoms during the scan period although long-term physiological or neural effects of infection remain largely unknown.

In summary, the current longitudinal analysis reported initial evidence of pandemic experience-related changes in brain emotion-related functions, where such changes reduced fear-associated learning activation in the PCC, premotor, hippocampus, visual cortices, brainstem, and thalamus regions. The effects of the pandemic experience were seen mainly in activation associated with low arousal stimulation, that is, context, safety cues, and extinguished fear cues. These findings may help identify brain mechanisms that underlie increased mental health disturbances caused by the pandemic. Yet, limited information about personal pandemic experiences impedes the identification of specific factors that contributed to these activation changes. While we speculate that the COVID-19 pandemic contributed to emotion activation changes, further comprehensive assessments of specific pandemic-related factors would be needed to further characterize changes in brain emotion functions.

## Data availability statement

The original contributions presented in the study are included in the article/Supplementary material, further inquiries can be directed to the corresponding author.

## Ethics statement

The studies involving humans were approved by the University of Toledo Institutional Review Board. The studies were conducted in accordance with the local legislation and institutional requirements. The participants provided their written informed consent to participate in this study.

## Author contributions

XW and HX: conceptualized the study, funding acquisition, and supervised the study. CP, AG, AZ, and C-HS: contributed to formal analysis. CP and AG: were involved in writing the original draft of the manuscript. CP, AG, C-HS, NC, AZ, XW, and HX: contributed to writing, reviewing, and editing of the manuscript. All authors contributed to the article and approved the submitted version.
