# Supplementary material for: Changes in fear-associated learning task brain activation over the COVID-19 pandemic period: a preliminary longitudinal analysis
Source: Front Psychiatry. 2023 Nov 22;14:1239697. doi: 10.3389/fpsyt.2023.1239697 (PMC10699136; doi:10.3389/fpsyt.2023.1239697)
Supplement: Supplementary file 1 [file Data_Sheet_1.docx]

**Supplementary materials**


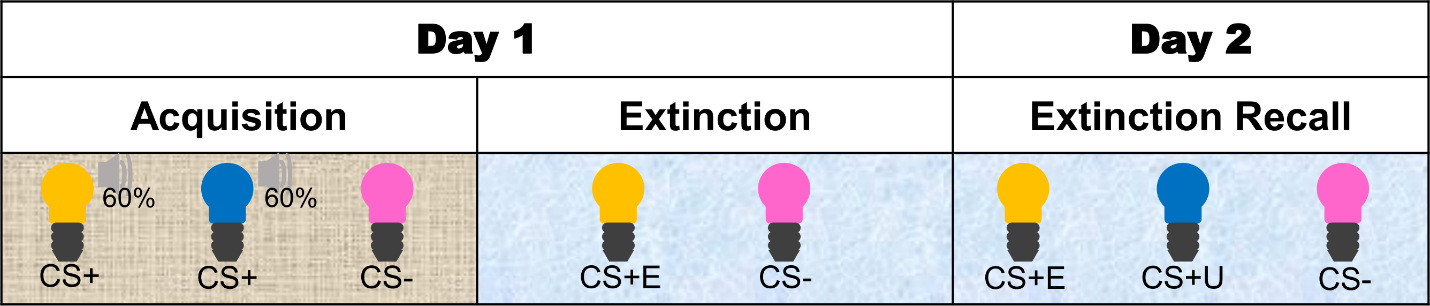


**Supplementary Figure 1.** Fear acquisition, extinction, and extinction recall. Fear acquisition contingencies were established on Day 1, followed by the extinction of the CS+ to form CS+E. On Day 2, extinction recall was tested using the presentation of the extinguished CS+, which was interleaved with CS- in the safety context.


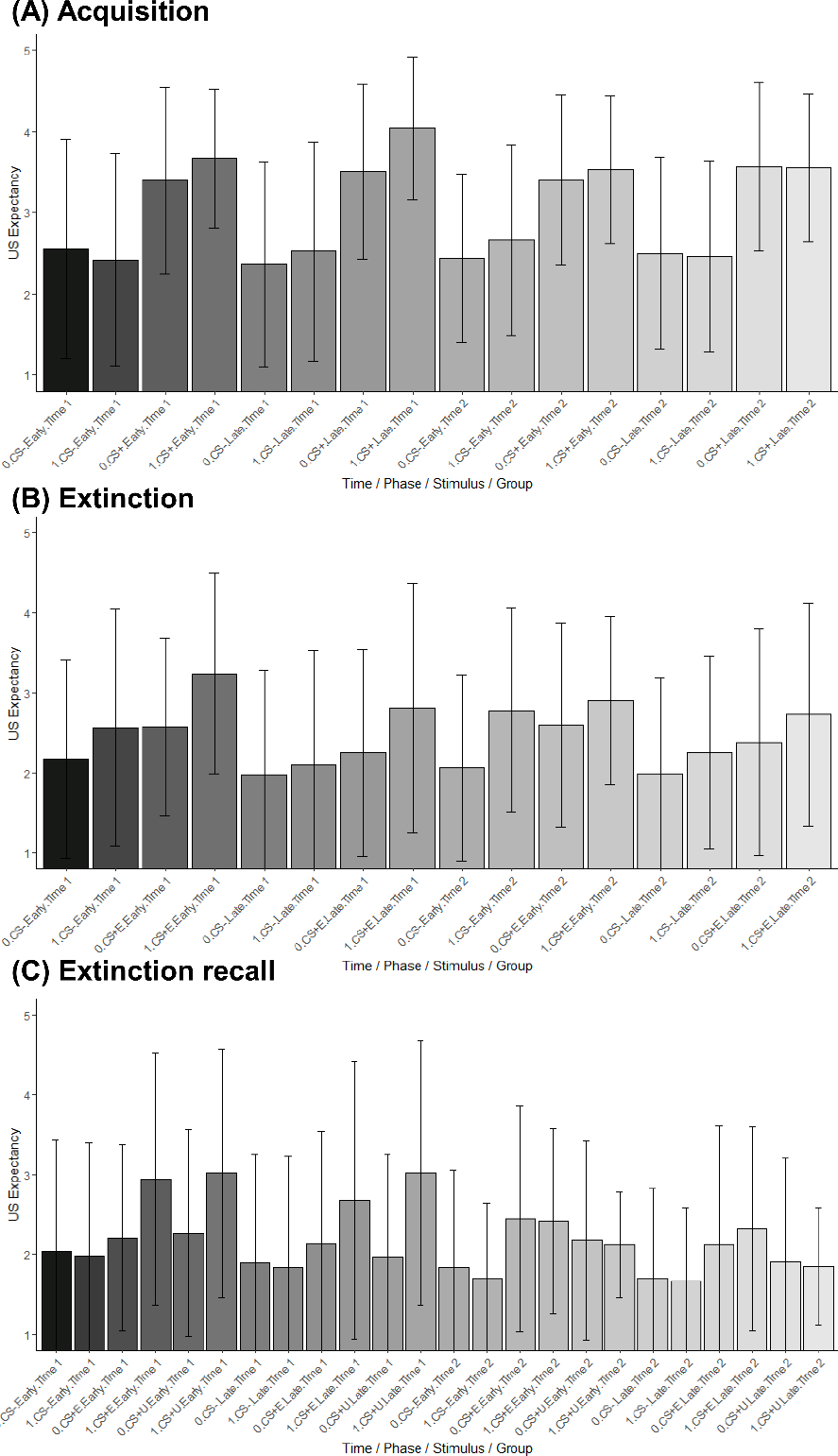


**Supplementary Figure 2.** Graphs depicting participants' US Expectancy during FALT: (A) fear acquisition; (B) extinction; and (C) extinction recall. Time 1 represents the baseline session; Time 2 represents the follow-up session. "Early" refers to the first half of each FALT phase; "Late" refers to the second half of each FALT phase. The labels "0" and "1" indicate the non-Pandemic and Pandemic groups, respectively.


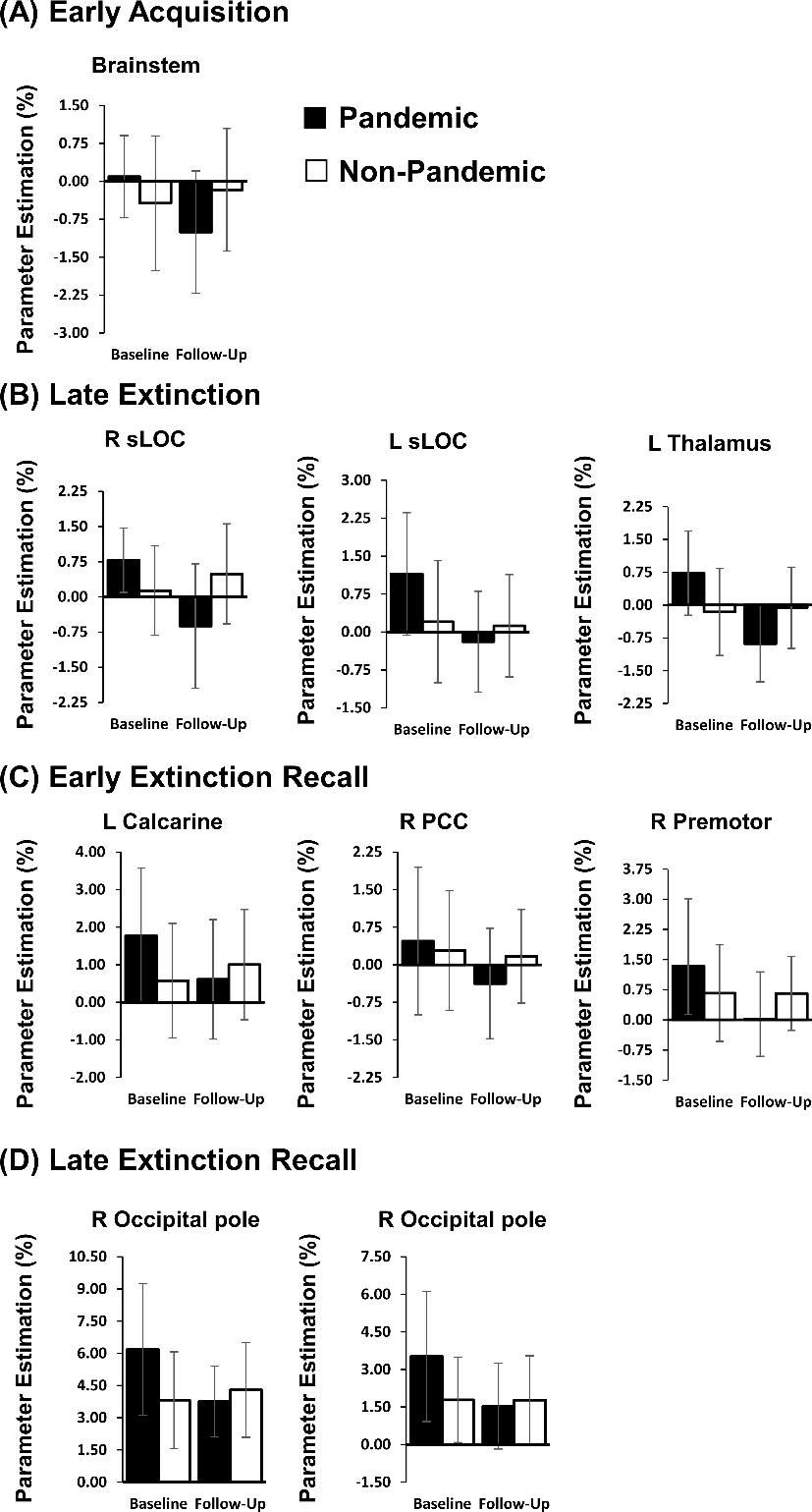


**Supplementary Figure 3.** Graphs depicting brain activation during FALT. Bar graphs show a Group (Pandemic vs. non-Pandemic) by Time (Baseline vs. Follow-up) comparison for extracted brain activation in (A) Early Acquisition, (B) Late Extinction, (C) Early Extinction Recall, and (D) Late Extinction Recall. "L" represents left; "R" represents right; "sLOC" stands for superior lateral occipital cortex; "PCC" stands for posterior cingulate cortex.
